# Brazilian Academy of Paediatric Otorhinolaryngology Task Force – lingual frenulum disorders in childhood – evidence-based recommendations^☆^

**DOI:** 10.1016/j.bjorl.2026.101762

**Published:** 2026-01-17

**Authors:** Juliana Alves de Sousa Caixeta, Debora Bressan Pazinatto, Rita Carolina Pozzer Krumenauer Padoin, José Faibes Lubianca Neto, Melissa Ameloti Gomes Avelino, Vitor Guo Chen, Trissia Maria Farah Vazzoler, Leticia Teixeira Castellano, Sulene Pirana, Carolina Sponchiado Miura, Edio Júnior Cavallaro Magalhães, Rodrigo Guimarães Pereira

**Affiliations:** a Academia Brasileira de Otorrinolaringologia Pediátrica, São Paulo, SP, Brazil; b Universidade Estatual de Campinas (UNICAMP), Campinas, SP, Brazil; c Serviço de Otorrinolaringologia Pediátrica, Hospital de Criança Santo Antônio da Santa Casa de Porto Alegre, Porto Alegre, RS, Brazil; d Universidade Federal de Ciências de Saúde de Porto Alegre, Porto Alegre, RS, Brazil; e Universidade Federal de Goiás, Goiás, GO, Brazil; f Universidade Federal de São Paulo, São Paulo, SP, Brazil; g Hospital Pequeno Príncipe, Curitiba, PR, Brazil; h Hospital das Clínicas Samuel Libanio, Pouso Alegre, MG, Brazil; i Universidade Estatual de São Paulo (USP-RP), Ribeirão Preto, SP, Brazil; j Hospital Municipal Souza Aguiar, Rio de Janeiro, RJ, Brazil

**Keywords:** Ankyloglossia, Lingual frenotomy, Lingual frenulum, Breastfeeding, Speech delay

## Abstract

**Objective:**

To provide evidence-based recommendations for the diagnosis, assessment of functional impact, and management of altered lingual frenulum in children.

**Methods:**

Task force members were educated on knowledge synthesis methods, including electronic database search, review and selection of relevant citations, and critical appraisal of selected studies. Articles written in English or Portuguese on ankyloglossia, or lingual frenotomy were eligible for inclusion. The American College of Physicians’ guideline grading system was used for critical appraisal of evidence and recommendations for therapeutic interventions.

**Results:**

There are several methods described in the literature for the evaluation of the lingual frenulum, and most of them lack internal or external validation. There are no data supporting a relationship between altered lingual frenulum and dysphagia, orofacial alterations, malocclusion, social limitations, obstructive sleep apnoea, or gastroesophageal reflux. According to the studies, an altered lingual frenulum does not cause speech delay; however, some studies with very low levels of evidence show that an altered lingual frenulum may make the pronunciation of some phonemes more difficult. In children with an altered lingual frenulum, lingual frenotomy may reduce maternal pain during breastfeeding. Therefore, in children with an altered lingual frenulum, lingual frenotomy may be a good therapeutic option when there is a complaint of pain during breastfeeding. There are several surgical techniques described in the literature for the release of the lingual frenulum, as well as different instruments that can be used for this purpose. There is no scientific evidence that one method is superior to another.

**Conclusion:**

Lingual frenectomy may improve maternal pain during breastfeeding and may be an option in selected cases of phonetic alterations.

## Highlights

• Most of methods to evaluate lingual frenulum lack internal or external validation.
• The diagnosis of submucosal lingual frenulum is controversial.
• Ankyloglossia does not cause speech delay, dysphagia, sleep apnoea or reflux.
• Lingual frenotomy may reduce maternal pain during breastfeeding.
• No surgical technique or instrument in frenectomy is superior to another.

## Introduction

Ankyloglossia, popularly known as “tongue-tie”, is a congenital condition characterized by the limitation of tongue mobility due to the presence of an altered lingual frenulum.^1^, ^2^, ^3^ The prevalence, in the literature, ranges from 0.02% to 20% in childhood, being difficult to measure this data correctly due to the absence of standardized diagnostic criteria that are easy to apply clinically.^2^^,^^4^, ^5^, ^6^ From an embryological point of view, ankyloglossia arises from the failure of apoptosis of the tissues that should separate the tongue from the floor of the mouth during fetal development, resulting in an anatomically altered frenulum.^7^

Over the years, there has been a significant increase in the diagnosis of ankyloglossia and performance of frenotomy. In the United States, between 1997 and 2012, the number of diagnosed cases grew by approximately 734%, while the number of procedures increased by about 870%. This rise was particularly pronounced after 2003, with an increase of about 4-times in diagnoses and 5-times in the number of frenotomies performed over the following nine years.^5^ This trend persisted: in the hospital setting alone, between 2012 and 2016, there was an increase of 110.4% in diagnoses, accompanied by a proportional growth in frenotomy rates.^8^ Similar data were observed in Canada, where, between 2002 and 2014, the diagnosis of ankyloglossia increased by 229% ‒ the prevalence rose from 6.86 to 22.6 cases per 1000 live births. In the same period, the number of frenotomies grew by 291%: from 3.76 to 14.7 per 1000 live births.^2^ In Australia, between 2006 and 2016, frenotomies in children aged 0–4 years increased by about 420%. The actual number, however, may be even higher, as the study did not include procedures performed in public hospitals or by dentists, especially using lasers.^9^

This trend cannot be explained solely by a higher prevalence of the condition, but is strongly associated with increased focus on breastfeeding, the rise in the number of lactation consultants, and the widespread dissemination of information on the topic through social media.^8^ In parallel with the growing clinical interest in ankyloglossia, a significant increase in scientific production on the subject has been observed. However, as occurs in rapid changes in medical practice, the generation of high-quality evidence has not kept pace with the growth of clinical interest, resulting in a scarcity of clear guidelines and difficulties in assessing the real benefits of frenotomy, due to methodological heterogeneity and biases in the available literature.^5^

This scenario has favoured the phenomenon of overdiagnosis of ankyloglossia, a concept that refers to the identification of a clinical condition that, although it may be present anatomically, may not cause significant functional repercussions and, therefore, does not require intervention. The hasty association between initial breastfeeding difficulties and ankyloglossia has led many families to choose frenotomy as an immediate solution, often without a careful and multidisciplinary clinical evaluation.^8^

The consequences go beyond the clinical scope: parents may experience anxiety, frustration with breastfeeding, and regret after procedures performed with the expectation of immediate improvement. There are also significant economic implications, such as the costs of multiple consultations, specialized evaluations, and the procedure itself.

The evaluation of the lingual frenulum, known as the “Tongue Test”, has been mandatory in all maternity hospitals in Brazil since 2014, following the enactment of Law nº 13002/2014.

## Objective

To make evidence-based recommendations for the diagnosis, assessment of functional impact, and management of the altered lingual frenulum in children.

## Method

The Brazilian Academy of Paediatric Otorhinolaryngology (ABOPe) selected several members to discuss the topics of this task force. Each author was responsible for reviewing the literature on each point addressed in this article, which was subsequently discussed among all members of the Task Force. The study reporter was responsible for drafting the text, which was then reviewed by the coauthors.

For the characterization of Levels of Evidence, the Methodological Guidelines Manual of the Brazilian Ministry of Health on the GRADE system was used. In the GRADE system, the assessment of evidence quality is performed for each outcome analysed for a given technology, using the available body of evidence.^10^ In GRADE, the quality of evidence is classified into four levels: high, moderate, low, and very low. These levels represent the confidence we have in the estimated effects presented, which is defined based on the study design (Table 1, Table 2). The randomized clinical trial is the most appropriate study design for questions related to interventions. When well-designed and properly conducted, the quality of evidence obtained from these studies starts as high, according to the GRADE system. When only observational studies are included, the quality of evidence starts as low. From the initial classification, criteria are defined, and the assessment of these aspects allows the level of evidence to be downgraded or upgraded. The factors responsible for downgrading the level of evidence are methodological limitations (risk of bias), methodological inconsistencies, use of indirect evidence, imprecision, and publication bias.

**Table 1:** Interpretation of the American College of Physicians’ guideline grading system (for therapeutic interventions).

Recommendation	Clarity of risk/benefit	Implications
Strong recommendation	Benefits clearly outweigh harms and burdens, or vice versa	**Patients:** Most would want course of action; a person should request discussion if an intervention is not offered
		**Clinicians:** Most patients should receive the recommended course of action
		**Policymakers:** The recommendation can be adopted as policy in most circumstances
Weak recommendation	Benefits closely balanced with harms and burdens	**Patients:** Many would want course of action, but some may not; the decision may depend on individual circumstances
		**Clinicians:** Different choices will be appropriate for different patients; the management decision should be consistent with patients’ preferences and circumstances
		**Policymakers:** Policymaking will require careful consideration and stakeholder input
No recommendation	Balance of benefits and risks cannot be determined.

**Table 2:** Recommendations (for therapeutic interventions) based on strength of evidence.

Recommendation and evidence of quality	Description of supporting evidence	Interpretation
**Strong recommendation**		
High-quality evidence	RCT without important limitations or overwhelming evidence from observational studies	Can apply to most patients in most circumstances without reservation
Moderate-quality evidence	RCT with important limitations or strong evidence from observational studies	Can apply to most patients in most circumstances without reservation
Low-quality evidence	Observational studies/case studies	May change when higher quality Evidence becomes available
**Weak recommendation**		
High-quality evidence	RCT without important limitations or overwhelming evidence from observational studies	Best action may differ based on circumstances or patients’ values
Moderate-quality evidence	RCT with important limitations or strong evidence from observational studies	Best action may differ based on circumstances or patients’ values
Low-quality evidence	Observational studies/case studies	Other alternatives may be equally Reasonable.
**Insufficient**	Evidence is conflicting, of poor quality, or lacking	Insufficient evidence to recommend for or against

RCT, Randomized Controlled Trial.

For the definition of recommendations, the following were considered: the overall quality of evidence, the balance between benefits and risks, resource use, equity, acceptability, and feasibility.

This guideline is not intended to replace the professional judgment of each physician in the evaluation of each patient, which is unique and involves many variables that cannot always be addressed in an article with academic purposes. This task force represents the assessment of an experienced team based on the best available literature on the subject.

## Results

### What is ankyloglossia?

The definition of ankyloglossia has been a subject of debate due to the absence of standardized diagnostic criteria and the subjectivity involved in assessments. Objectively, ankyloglossia is the term used to define an altered lingual frenulum, thus representing an anatomical definition. The classical form, called anterior ankyloglossia, is the presence of a lingual frenulum that extends to or near the tip of the tongue, restricting its mobility ‒ an anatomical alteration generally evident upon clinical inspection.^1^^,^^5^^,^^11^

The concept of posterior ankyloglossia, however, remains controversial, with no consensus among specialists, as demonstrated in the *Clinical Consensus Statement: Ankyloglossia in Children*, published in 2020 by the American Academy of Otorhinolaryngology.^1^ While some panel members consider the term to refer to a frenulum inserted in the posterior portion of the tongue, others use it to describe a submucosal lingual frenulum, identifiable only through specific maneuvers. There are also those who question the existence of posterior ankyloglossia as a distinct anatomical entity, suggesting that the term be abandoned due to its diagnostic subjectivity.^1^^,^^12^

### What are the methods for evaluating the lingual frenulum in children?

Several methods have been proposed for the evaluation of the lingual frenulum in children, with considerable variations regarding the anatomical and functional criteria considered, as well as the subjectivity of the assessment and its clinical applicability. Here, we present some of the main tools described in the literature.

One of the most frequently cited methods in the literature is the Hazelbaker Assessment Tool for Lingual Frenulum Function (HATLFF).^13^ This protocol, published in 1994, evaluates both anatomical and functional aspects of the lingual frenulum and is mainly used in clinical contexts focused on breastfeeding. The HATLFF is a 12-item scale, with 10-points for the assessment of the anatomical aspect of the frenulum and 14-points for the functional aspect. A score above 24 indicates normal function and anatomy. According to this scale, frenotomy is necessary for symptomatic ankyloglossia if the appearance score is below 8, or if the function score is below 11 combined with an appearance score lower than 10. For perfect function scores, regardless of appearance, frenotomy would not be recommended. A study conducted by Amir et al., in 2006, investigated the reliability of this tool and identified limitations in the standardization of functional assessment, as well as the need for specific training of the professionals applying it.^13^^,^^14^

In 1999, Kotlow published a classification that evaluates the free tongue length, measured from the tip of the tongue to the insertion of the frenulum. A length greater than 16 mm is considered normal, and the classification includes four degrees of alteration, with grade IV being considered complete ankyloglossia (length ≤ 3 mm).^11^ Although it is an objective method of evaluation, its main limitations include the absence of assessment of functional aspects and the possible difficulty in obtaining precise measurements in very young children.

The Coryllos classification, from 2004, includes four types of lingual frenulum, with type I being the most altered (frenulum inserted into the alveolar groove, resulting in a heart-shaped tongue) and type IV, in which the frenulum is not visible but palpable, with fibrous or submucosal insertion. This classification includes what is described in the literature as the submucosal frenulum.^15^ It is worth noting that the existence of the submucosal frenulum is questioned by several authors. In a prospective cohort study, the prevalence of a “submucosal frenulum” in asymptomatic children was about 59%.^16^

In Brazil, in 2012, the Tongue Test, described by Martinelli et al., was developed. It proposes a systematized approach for neonatal screening, considering both anatomical and functional aspects.^17^ This assessment is divided into four sections, including the evaluation of tongue position at rest, the tendency of tongue positioning during crying, the shape of the tongue during crying, and the anatomical evaluation of the lingual frenulum. This is a method that contains several subjective aspects that may influence the final score, and since it includes subitems within each section, it requires training for its proper application.

In 2015, another assessment tool was published ‒ the Bristol Tongue Assessment Tool (BTAT) ‒ developed in the United Kingdom by Ingram et al.^18^ It is a rapid application protocol, derived from the HATLFF and based on simple scoring, aimed at identifying ankyloglossia in newborns. The exam is considered normal when it reaches 8 points, altered when the score is below 3, and doubtful between 4 and 5 points. This is the tool indicated in the technical note of the Brazilian Ministry of Health as the reference for evaluation in children. There is, however, no justification provided in that note for the choice of this specific instrument. In 2019, the Tongue-tie and Breastfed Babies Assessment Tool (TABBY) was also published, consisting of a graphical representation of the BTAT. Its main objective is to make the assessment more visual and practical, following the same scoring system as the BTAT.^19^

**Fig. 1:**
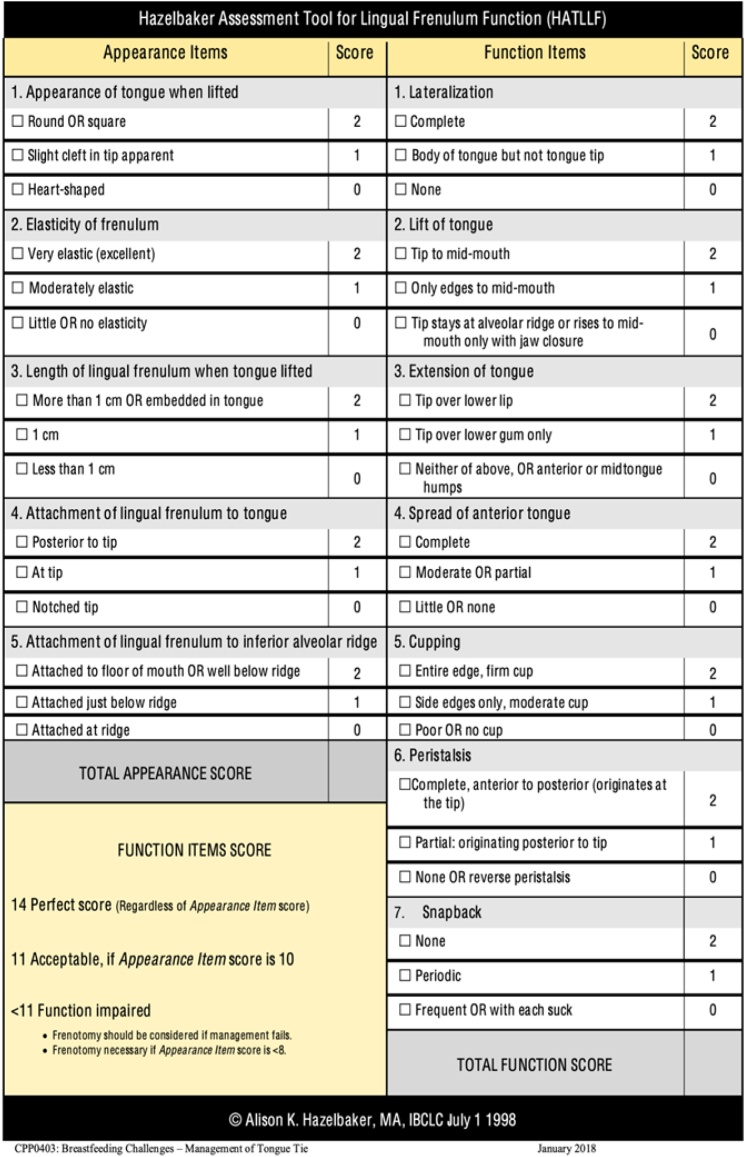
Hazelbaker assessment tool for lingual frenulum function. From: https://www.researchgate.net/publication/343677587_Prevalence_of_neonatal_ankyloglossia_in_a_tertiary_care_hospital_in_Spain_a_transversal_cross-sectional_study.

**Fig. 2:**
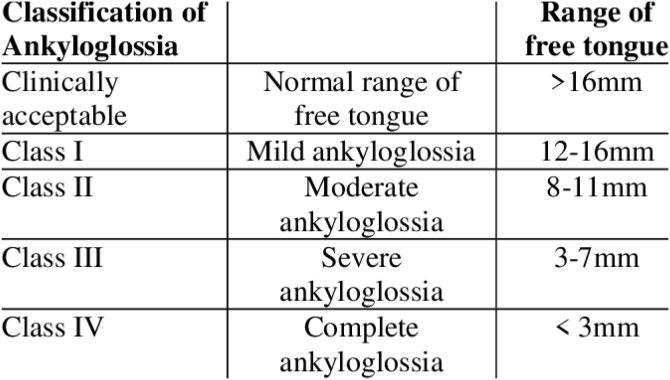
Kotlow’s classification of ankyloglossia. From: https://www.researchgate.net/publication/348957271_Tongue_tie_A_case_report.

**Fig. 3:**
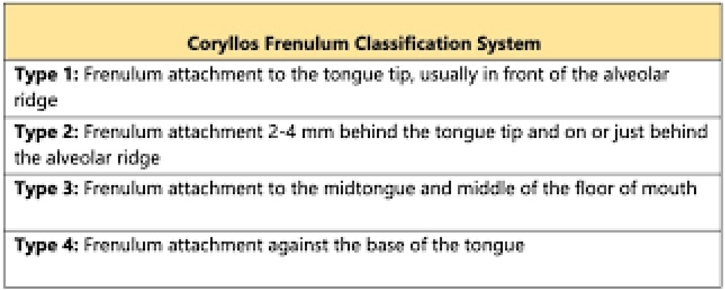
Coryllos method (Without validation to Portuguese). From: https://www.ncbi.nlm.nih.gov/books/NBK482295/figure/article-17608.image.f1/.

**Fig. 4:**
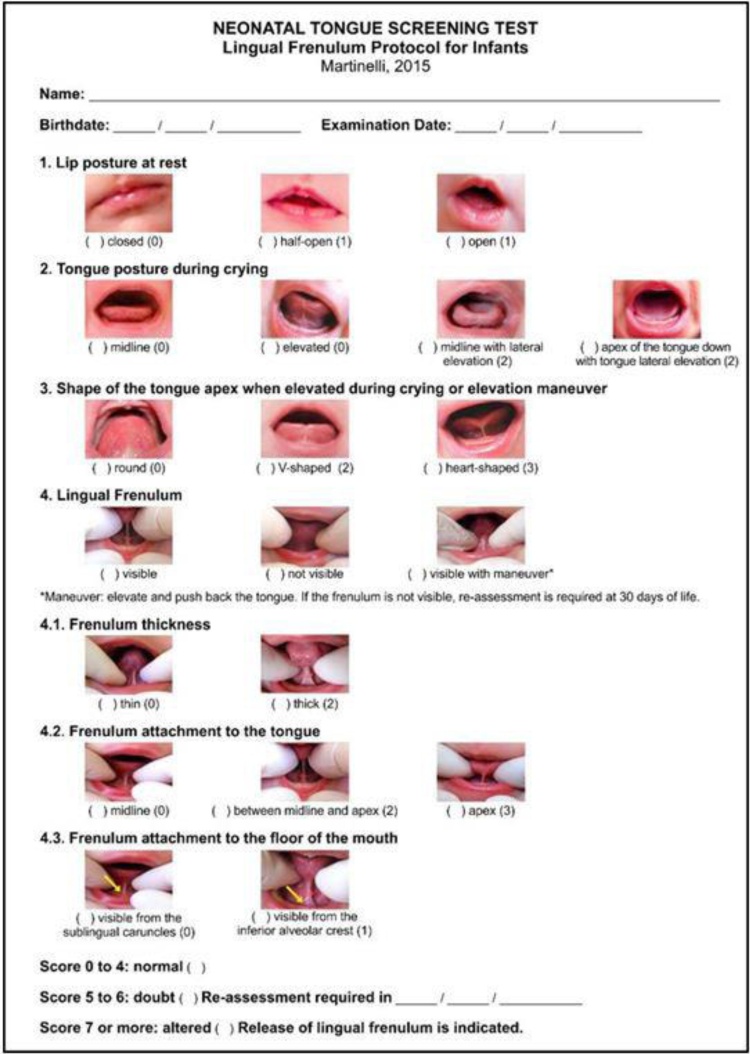
Lingual frenulum protocol for infants – Martinelli et al.^75^. From: https://www.researchgate.net/publication/331328592_Association_between_ankyloglossia_and_breastfeeding.

It is important to emphasize that none of the methods for evaluating the lingual frenulum described in the literature have undergone internal or external validation studies. A study published in 2021 evaluated 147 newborns using both the Martinelli et al. method and the BTAT, on the same occasion. The results showed that the prevalence of ankyloglossia was higher when using the Martinelli protocol, with a statistically significant difference.^15^ A meta-analysis published in 2022 showed that the prevalence of the diagnosis of altered lingual frenulum may vary according to the method used, with the Coryllos method leading to a higher prevalence of diagnosis. Another interesting finding from that same article is that, progressively, more recent publications show a higher prevalence of this diagnosis when compared to studies from previous years or decades (Fig. 1, Fig. 2, Fig. 3, Fig. 4).^20^

The tools described here for the evaluation of the lingual frenulum are presented in Appendix B.

### What are the functional implications of an altered lingual frenulum in children?

#### Altered lingual frenulum and obstructive sleep apnoea

There are several risk factors for Obstructive Sleep Apnoea (OSA) in children that are well described in the literature. A correlation has been observed between obesity, mucopolysaccharidosis, adenotonsillar hypertrophy, craniofacial abnormalities, Down syndrome, and a higher prevalence of OSA.^21^ Recently, the presence of a short lingual frenulum has been associated with the diagnosis of sleep apnea,^22^ supported by the concept that the tongue is a key muscle in maintaining the patency of the upper airways, playing an essential role in oropharyngeal support.^23^ According to this concept, at birth, the tongue naturally rests on the palate, and its active participation in the functions of sucking, swallowing, and chewing stimulates the intermaxillary synchondrosis, a fundamental process for normal growth of the face and oral cavity and for maintaining the nasal breathing pattern, highlighting the complex relationship between tongue activity and craniofacial development.^24^

Some authors propose that the restriction imposed by the frenulum limits tongue mobility, which impairs the proper growth of the palatal arch and the maxilla. A narrow palatal arch, in turn, has been associated with a higher risk of OSA due to the reduced space of the upper airways.^22^, ^23^, ^24^

The association between altered lingual frenulum and OSA, so far, is based on data from retrospective studies with limited methodology.^25^^,^^26^ At the same time, there are studies indicating the opposite: that anterior tongue attachment may, to some extent, prevent posterior tongue collapse during sleep. Thus, performing frenulum release procedures could theoretically increase the risk of posterior tongue collapse and, consequently, worsen OSA in some cases.^5^^,^^27^

A systematic review published in 2024 concluded that there is an association between ankyloglossia and OSA in children. This review included six studies: three cross-sectional studies, two retrospective cohorts, and no clinical trials. Only two studies used polysomnography, which is the gold standard for the definition of OSA, with diagnostic criteria, while the other studies used questionnaires or medical record data. The methodological flaws of this review and the risk of bias limit the interpretation of the results.^25^ Furthermore, OSA is a complex and multifactorial condition; the presence of an altered lingual frenulum alone should not be considered a determinant for this association.

**There is an association between OSA and altered lingual frenulum – Level of Evidence: Very Low**

**Lingual frenotomy is indicated for the treatment of OSA – Level of Evidence: Low – Recommendation: Not Recommended**

#### Altered lingual frenulum and dental and orofacial changes

Two pathophysiological mechanisms have been suggested to justify a possible association between an altered lingual frenulum and the development of malocclusion: the first involves restriction of tongue posture, which would reduce the pressure exerted on the palate, favouring the formation of a high-arched palate or posterior crossbite; the second proposes that functional alterations in chewing and phonation could generate muscular compensations capable of influencing mandibular growth vectors.^28^

This association is based on case reports, case series, retrospective studies, and cross-sectional studies.^24^^,^^26^^,^^27^^,^^29^ Furthermore, in many studies, the method for evaluating the lingual frenulum was not adequately described, which may have led to inconsistencies in diagnosis and a high risk of bias.^28^^,^^30^ It is worth noting that children with ankyloglossia may present malocclusion; however, the main risk factors remain being genetic inheritance, deleterious oral habits (such as non-nutritive sucking or atypical swallowing), and mouth breathing.

A systematic review and a systematic review with meta-analysis published in 2024 evaluated the relationship between ankyloglossia and malocclusion/facial development and concluded that there is no sufficient scientific evidence to indicate frenotomy for the purpose of preventing or treating malocclusion.^25^^,^^26^ The quality of evidence of the articles included in these reviews was considered low or very low. No randomized clinical trials were identified, and there is high methodological heterogeneity among the included studies.^26^^,^^27^

To clarify the possible association between ankyloglossia and changes in dental arch development ‒ as well as the role of frenotomy in preventing these changes - studies with greater methodological robustness are needed. Data such as objective and reproducible evaluation measures are essential to assess this correlation, as well as prospective and long-term follow-up of these patients.

**There is an association between altered facial growth and altered lingual frenulum – Level of Evidence: Very Low**

**There is an association between malocclusion and altered lingual frenulum – Level of Evidence: Very Low**

**Lingual frenotomy is indicated for the treatment or prevention of facial alterations – Level of Evidence: Low – Recommendation: Not Recommended**

**Lingual frenotomy is indicated for the treatment or prevention of malocclusion – Level of Evidence: Low – Recommendation: Not Recommended**

#### Altered lingual frenulum and dysphagia

The altered lingual frenulum has been widely studied in the context of breastfeeding. However, its clinical relevance after this period, in relation to swallowing and dysphagia in older children, remains controversial. Observational studies and case reports have suggested that restriction of tongue base movement, especially in cases of posterior ankyloglossia, could impact the pharyngeal phase of swallowing.^31^ A retrospective analysis of 226 patients who underwent frenotomy identified improvement in aspiration in 43% of cases that had a swallowing study performed before and after the procedure. Although the data indicate a possible benefit of frenotomy, the authors emphasize that most patients had other comorbidities that could contribute to dysphagia, making the analysis of results more complex.^32^ The relationship between ankyloglossia and dysphagia becomes even more challenging to assess in the coexistence of other complex clinical conditions, such as cerebral palsy, prematurity, or genetic syndromes, making it difficult, if not impossible, to distinguish whether the cause of dysphagia is the anatomical alteration of the lingual frenulum or the underlying condition.^33^

Baxter et al. prospectively evaluated 37 children who underwent laser frenotomy and concluded that there was significant improvement in feeding with solids among the children. This study did not include a control group, did not use any method for swallowing assessment (only parental reports), and frenotomy was not the only intervention performed on the patients, who simultaneously underwent myofunctional exercises. The study’s limitations require caution in generalizing the data.^33^ Similarly, a case series reported immediate improvement in tongue mobility, speech, and feeding in five children after surgical tongue release.^34^

A 2023 literature review analysed 26 studies with data from more than 1200 patients, suggesting that ankyloglossia may be associated with a range of symptoms in older children, including feeding difficulties and dysphagia. However, the authors also emphasize that the available data are still inconclusive since the level of evidence of the studies evaluated is low or very low.^35^

In summary, the findings to date, although weak, suggest that the correction of ankyloglossia may be beneficial in selected cases, provided it is preceded by a comprehensive clinical evaluation with the participation of a multidisciplinary team. The challenge remains in distinguishing symptoms attributable to ankyloglossia from those resulting from other aetiologies, reinforcing the need for prospective studies with adequate control of confounding variables and longitudinal follow-up of functional outcomes.

**There is an association between dysphagia and altered lingual frenulum – Level of Evidence: Very Low**

**Lingual frenotomy is indicated for the treatment of dysphagia – Level of Evidence: Low – Recommendation: Not Recommended**

#### Altered lingual frenulum and gastroesophageal reflux

The proposed mechanism for the possible relationship between ankyloglossia and symptoms of Gastroesophageal Reflux (GER) is hypothetical: a short lingual frenulum would lead to inefficient sucking during breastfeeding, resulting in air swallowing (aerophagia), gastric distension, and consequent functional regurgitation.^36^ This hypothesis would explain the improvement in GER symptoms after frenotomy described in some scientific articles. One of the greatest challenges in evaluating GER in childhood is that the diagnosis is essentially clinical. The lack of questionnaires or clinical scores with high clinical correlation, the absence of a gold-standard diagnostic test, and the difficulty in distinguishing GER from Gastroesophageal Reflux Disease (GERD) in childhood ‒ particularly in retrospective studies ‒ limit the accuracy of this diagnosis. Especially in infants, physiological GER is even more prevalent, and the diagnosis of GERD requires the assessment of other conditions such as dysphagia and cow’s milk protein allergy.^37^

Ghaheri et al. prospectively evaluated 237 infants aged between 0- and 12-weeks who underwent diode laser frenotomy. The method used to assess GER symptoms was the I-GERQ-R (Revised Infant Gastroesophageal Reflux Questionnaire), administered preoperatively, one week, and one month after frenotomy. A significant improvement was observed in the scores reported by the mothers, with the most pronounced improvement among infants who underwent the procedure between 5- and 8-weeks of age.^38^

A systematic review published in 2025 included seven studies (six prospective cohorts and one clinical trial) and concluded that lingual frenotomy may improve GER symptoms in children.^39^ None of the studies included in this review used objective methods for GER assessment, such as pH monitoring or multichannel intraluminal impedance. Although improvements in reflux scores were reported after frenotomy, the absence of adequate control groups, the use of inappropriate measurement instruments, and the high risk of bias limit the interpretation of this study’s results. The only clinical trial included in this review presents some limitations, such as selection bias and the fact that GER assessment was not the primary outcome of the study.

It is worth noting that GER, especially in infants, improves with age, making it difficult to attribute clinical improvement exclusively to the procedure.^37^^,^^39^ Future randomized clinical trials using validated measures, both instrumental and self-reported, are needed to better elucidate the efficacy of frenotomy in resolving GER symptoms in infants with ankyloglossia.

**There is an association between gastroesophageal reflux and altered lingual frenulum – Level of Evidence: Low**

**Lingual frenotomy is indicated for the prevention or treatment of gastroesophageal reflux – Level of Evidence: Low – Recommendation: Not Recommended**

#### Altered lingual frenulum and social implications

Some authors suggest that untreated ankyloglossia could be associated with functional and psychosocial limitations that tend to manifest during adolescence and adulthood. Difficulties are suggested in actions such as licking the lips, consuming foods like ice cream, kissing, playing wind instruments, maintaining oral hygiene, or controlling salivation. Although these restrictions may seem subtle from a clinical standpoint, they could negatively impact quality of life, especially in social contexts where these functions have symbolic, cultural, or interpersonal relevance.^40^

Most studies evaluating the benefits of ankyloglossia correction for social concerns include heterogeneous populations, composed of children and adults with a wide age range. These are generally low-quality studies, mostly retrospective and with a small number of participants.^41^

Two case series considered specific aspects, such as the influence of ankyloglossia on kissing, playing wind instruments, excessive salivation, and oral hygiene.^40^^,^^42^ These investigations, however, were limited by the lack of pre-procedure information on these skills and by the absence of detailed description of the evaluation methods used. Furthermore, the patients were selected through retrospective chart review or based on their presentation at otorhinolaryngology clinics, and social concerns were measured only as secondary outcomes.

A randomized clinical trial evaluated tongue mobility using two different surgical techniques for the treatment of ankyloglossia and found that both significantly improved tongue mobility.^43^ In a cohort study, individuals with untreated ankyloglossia had the worst tongue mobility, followed by children with treated ankyloglossia, and finally those with no history of ankyloglossia, around 6-years of age.^44^ However, there are no standardized definitions of tongue mobility nor established norms for what constitutes “normal” mobility. Standardization would allow comparability between studies and enable data aggregation in future meta-analyses.

**An altered lingual frenulum may have social/functional impact on an individual’s life – Level of Evidence: Very Low**

**Lingual frenotomy is indicated for the prevention or treatment of possible social or functional implications – Level of Evidence: Low – Recommendation: Not Recommended**

#### Altered lingual frenulum and impact on speech development

Speech and language are distinct concepts. Speech is the physical means of transmitting oral language, while language is the underlying cognitive system that organizes and gives meaning to human communication.

Speech refers to the motor act of producing articulated vocal sounds, involving the coordination of the speech organs (lips, tongue, palate, larynx, etc.) to generate the acoustic signal that conveys the verbal message. It is, therefore, a physical and motor phenomenon, dependent on the anatomical and functional integrity of the nervous system and the vocal tract. Speech disorders include alterations in articulation, fluency (such as stuttering), and voice.^45^^,^^46^ Language, on the other hand, is a broader cognitive system responsible for the ability to understand, organize, and express ideas through symbols (words, gestures, signs), following syntactic, semantic, and pragmatic rules. It can be expressed through different modalities, including speech, writing, and sign language, and does not depend exclusively on vocal production.^45^

Historically, alteration of the lingual frenulum has been associated with problems in speech clarity; for this reason, the term “tongue-tie” was used to describe people with low speech intelligibility. There are no studies demonstrating a relationship between altered lingual frenulum and speech delay. The hypothesis is that individuals with ankyloglossia would be unable to protrude the tip of the tongue beyond the mandibular incisors or to touch the palate, causing impairment in the production of sounds that depend on this movement, such as /l/, /r/, /t/, /d/, /z/, /s/, /n/, and /th/.^47^ Other functional movements, such as tongue lateralization, tongue spreading, cupping, and rapid tongue retraction, would also be restricted. It is important to emphasize that tongue elevation, not protrusion, is the crucial movement for speech; in other words, even children with restricted tongue protrusion can produce phonemes correctly.^48^

An important consideration regarding the impact of altered lingual frenulum on speech is that the phonemes present in each language are different. In Brazil, a study published in 2011 evaluated 53 individuals aged 2–33 years who underwent lingual frenotomy, and another study published in 2013 evaluated 13 patients aged 7–43 years who underwent speech assessment six months after lingual frenotomy combined with speech therapy.^49^^,^^50^ Both concluded that lingual frenotomy has a beneficial effect on speech. However, the level of evidence of these studies is very low (lack of sample size calculation, small sample, heterogeneous samples, and inadequate outcome assessment methods).

Chinnadurai et al. conducted a systematic review published in 2015 showing that studies evaluating the effect of lingual frenotomy on speech improvement present low-quality evidence with a high risk of bias, including differences in outcome assessments.^41^ In 2020, Wang et al. published a systematic review including 16-articles evaluating whether: (a) Patients diagnosed with ankyloglossia or short lingual frenulum had a higher risk of speech and articulation disorders, and (b) Surgery could improve patients’ speech dysfunction. Among the 16-articles, three are clinical trials, four are prospective cohort studies, six are retrospective cohort studies, one is a cross-sectional study, and two are case series; of these, six used some method to assess speech articulation. This review discusses the low quality of the studies, methodological gaps, and high risk of bias, and concludes that it is not possible to establish an association between altered lingual frenulum and speech alteration, nor to attribute speech improvement to surgery.^51^ Some authors discuss that individuals with altered lingual frenulum may be able to produce speech sounds adequately through compensatory movements.^44^^,^^51^, ^52^, ^53^

Nonetheless, some observational studies and scoping reviews report a possible association between moderate to severe ankyloglossia and articulatory difficulties, especially in subgroups of patients, such as children with significant functional tongue limitation. In these cases, there may be sound distortion, reduced intelligibility, and specific difficulties in producing sibilants or other phonemes that depend on more complex tongue movements.^48^

**An altered lingual frenulum may impact a child’s speech – Level of Evidence: Low**

**Lingual frenotomy is indicated for the prevention of speech alterations – Level of Evidence: Low – Recommendation: Not Recommended**

**Lingual frenotomy is indicated for the treatment of speech alterations – Level of Evidence: Low – Recommendation: Not Recommended**

#### Altered lingual frenulum and impact on breastfeeding

The World Health Organization recommends that children be exclusively breastfed until six months of age, with breastfeeding continuing up to two years or longer. The prevalence of exclusive breastfeeding among children under 6-months in the country was 45.8%, according to the National Survey on Infant Feeding and Nutrition (ENANI) published in 2021.^54^ Alteration of the lingual frenulum has been reported in several studies as a limiting factor for breastfeeding, since ankyloglossia can hinder proper latch onto the maternal breast, leading to non-nutritive sucking, aerophagia, pain during breastfeeding, and nipple fissures.^44^^,^^55^^,^^56^

Three cohort studies evaluated the relationship between ankyloglossia and exclusive breastfeeding at six months of age. Two of these studies showed that the rate of children exclusively breastfed until six months was not different between those with and without altered lingual frenulum.^57^ Another study evaluated 225 children who were followed regarding exclusive breastfeeding during the first six months of life. Asymptomatic patients with altered lingual frenulum (assessed by BTAT) were included and compared with children with normal examination. Patients diagnosed with ankyloglossia were breastfed for an average of 4.5-months, while patients with normal frenulum were breastfed for an average of 5.4-months (*p* < 0.05). The quality of these studies is considered very low.^58^

A clinical trial published in 2011 evaluated 58 newborns with ankyloglossia and breastfeeding difficulties, comparing 30 children who underwent lingual frenotomy with 28 controls. It showed that, after two weeks of the intervention, maternal pain was reduced in both groups, with a greater reduction in the intervention group (*p* < 0.05).^55^

Another clinical trial evaluated 107 newborns with ankyloglossia and breastfeeding difficulties, comparing 55 patients who underwent frenotomy with 52 controls. This study showed improvement in breastfeeding difficulty, assessed by the Infant Breastfeeding Assessment Tool, and in maternal pain after frenotomy. The follow-up period was five days after the intervention.^59^

A systematic review published in 2022 included six studies, four randomized clinical trials and two prospective cohort studies. For the meta-analysis, only the clinical trials were included. This review concluded that lingual frenotomy had a positive effect on the subjective complaint of breastfeeding difficulty and that maternal pain during breastfeeding was reduced. The authors reported that the review, which included 329 children for data analysis, has limitations, such as the small sample size of some studies and heterogeneity among studies (I² > 90%).^60^

Another review published in the Cochrane Database of Systematic Reviews, also in 2022, included five randomized clinical trials with a total of 302 patients. The primary objective of the study was to determine whether frenotomy is effective in improving breastfeeding in children under 3-months of age.^61^ There was improvement in the complaint of breastfeeding difficulty after the procedure and in nipple pain. It was not possible to evaluate whether frenotomy increased adherence to breastfeeding, as only one of the studies followed patients for more than 3-months. The study’s limitations include the small sample size (with only one study evaluating more than 100 patients) and the method of assessing breastfeeding difficulty (two of the five studies used subjective methods and three studies used scales).

The results of these meta-analyses should be interpreted with caution due to the small number of patients included the inclusion of studies that assessed the lingual frenulum and breastfeeding difficulty using different methods, and the short follow-up period. However, considering the relative effect estimate of the intervention as well as the risk estimate of lingual frenotomy, this group considers that the procedure should be considered in children with altered lingual frenulum when there is a report of pain during breastfeeding.

**An altered lingual frenulum may impact breastfeeding difficulty – Level of Evidence: Moderate**

**Lingual frenotomy may be an option for managing breastfeeding-related pain – Level of Evidence: – Recommendation: Recommended**

#### What is lingual frenotomy and what surgical techniques have been described?

In the literature, the terms *frenulotomy*, *frenotomy*, *frenulectomy*, and *frenuloplasty* are used to describe the surgical procedures employed to correct an altered lingual frenulum. The most frequently cited procedure is lingual frenotomy, which consists of the incision or division of the lingual frenulum.^5^^,^^62^, ^63^, ^64^ Although many articles use these terms interchangeably to describe technically different procedures, frenectomy is generally considered distinct from frenotomy, as it involves the complete excision of the frenulum and is therefore a more invasive technique, typically used when the frenulum is thicker or fibrotic.^34^^,^^63^^,^^65^^,^^66^ *Frenuloplasty*, in turn, is a more elaborate technique that involves the release and repositioning of tissues to lengthen the lingual frenulum and minimize recurrence.^43^^,^^64^^,^^67^ The variability in terminology and techniques found in the literature is, without a doubt, a source of confusion when attempting to perform clear comparative analyses.

The conventional lingual frenotomy is considered a quick and low-risk procedure. It is frequently indicated for newborns, as they present with a thin and poorly vascularized frenulum, resulting in minimal discomfort and bleeding.^5^^,^^7^^,^^68^ The procedure consists of elevating and stabilizing the tongue (which can be performed with the fingers or with a grooved retractor), followed by an incision with fine surgical scissors along the frenulum, close to the tongue, taking care not to injure the ducts of the submandibular glands.^12^^,^^62^^,^^64^ The use of sutures depends on the surgeon’s experience and is particularly useful to assist with haemostasis.

In addition to the various surgical techniques described in the literature, the use of different instruments is another point of discussion. The use of cold instruments for performing frenotomy includes scalpel blades and surgical scissors, while the use of thermic instruments includes electrocautery and different types of lasers.

The effectiveness of local anaesthetics remains a controversial topic in the literature, and their use varies among surgeons. A study published in 2022 showed that 29% of otolaryngologists use topical lidocaine for this procedure.^68^ Other studies describe the use of sucrose drops, tetracaine, and benzocaine. A randomized clinical trial on topical anaesthesia for conventional frenotomy performed in-office in infants aged 0 to 3-months concluded that the available topical anaesthesia options are not effective, making their use unnecessary.^69^

There is no consensus regarding essential postoperative care. Some authors suggest that myofunctional rehabilitation improves tongue mobility and ensures long-term treatment success by preventing adhesions and recurrence.^47^^,^^63^^,^^70^

#### Comparison between the different surgical techniques described in the literature

Yousefi et al. conducted a randomized clinical trial directly comparing “Z” frenuloplasty with conventional frenotomy and concluded that there was no difference between the two techniques for the outcome studied.^71^ These same authors consider that “Z” frenuloplasty may yield superior results when performed to treat speech problems, since this procedure is usually performed in older children and involves various techniques (with variations of flaps and with or without partial myotomy of the genioglossus muscle). However, it is important to note that the association between speech alterations and ankyloglossia remains controversial.

Frezza et al. published a narrative literature review in 2022 including 14 articles: one clinical trial, four prospective cohort studies, one case-control study, three cross-sectional studies, and four retrospective studies.^47^ The authors concluded that there is a preference among professionals for the use of diode laser, which offers advantages compared to the conventional procedure. Although it is a review article, this conclusion is supported solely by the authors’ opinion, since the study has many methodological limitations, and the results are limited to a single page of supplementary material consisting of a table describing the included studies.

Khan et al., in a systematic review published in 2020, compared different surgical treatment techniques for ankyloglossia.^64^ This study, which included 35-articles, seven of which were randomized clinical trials, concluded that conventional frenotomy, laser frenotomy, and *frenuloplasty* are all good options for the surgical treatment of ankyloglossia when considering their impact on breastfeeding. The authors state that there is no evidence that one technique is superior to the others. All techniques were considered safe and effective procedures for treating an altered lingual frenulum, but the articles demonstrating the benefits of conventional frenotomy were generally of higher quality compared to other frenotomy methods. Additionally, conventional frenotomy was more commonly performed without anaesthesia compared to *frenuloplasties* or procedures using electrocautery and lasers. There was no evidence of benefits for “Z” *frenuloplasty* or laser *frenotomy* for the treatment of ankyloglossia in the paediatric population compared to conventional frenotomy.

This finding is in accordance with publications by the American Academy of Paediatrics (AAP) and the American Academy of Otorhinolaryngology – Head and Neck Surgery (AAO-HNS), which indicate that there are no differences between the various techniques or the different instruments used for the surgical treatment of altered lingual frenulum, and that the choice of instrument is a matter of personal preference.^1^^,^^72^^,^^73^

**Laser is superior to other instruments for performing lingual frenotomy – Level of Evidence: Low**

**There is one surgical technique superior to others for the treatment of altered lingual frenulum – Level of Evidence: Low**

#### Considerations on the limitations and risks associated with the surgical treatment of altered lingual frenulum

There are no well-designed studies that establish the complication rate of lingual frenotomy. Solis-Pazmino et al. reported the case of an infant who developed a mucocele after laser frenotomy and, in a narrative literature review, described the most common complications as: immediate post-procedure feeding/breastfeeding difficulties, bleeding, and bleeding-related complications such as hypovolemic shock and hematoma.^74^ Infectious complications reported in the literature included Ludwig’s angina, infected hematoma, surgical site infection, infected cyst, and submandibular abscess. Complications related to injury of structures adjacent to the lingual frenulum may also occur, such as salivary gland injury, mucocele, and massive submandibular edema due to Wharton’s duct obstruction, potentially leading to airway obstruction. These authors also cited reports of two infants with Pierre Robin sequence who developed airway obstruction following frenotomy.

One of the main points to be discussed is the overestimation of the contribution of an altered lingual frenulum to the various conditions addressed here. A proper clinical evaluation should consider differential diagnoses that may affect breastfeeding, swallowing, speech, sleep, and the patient overall. Considering an altered lingual frenulum as the primary or sole cause of different symptoms can lead to underdiagnosis of relevant clinical conditions, neglecting or delaying the correct diagnosis for patients.

## Conclusion

Most of methods to evaluate lingual frenulum lack internal or external validation, and the diagnosis of submucosal lingual frenulum is controversial. Literature review shows that the altered lingual frenulum does not cause orofacial alterations, malocclusion or sleep apnoea. The altered lingual frenulum does not cause speech delay, dysphagia or reflux. Lingual frenotomy may reduce maternal pain during breastfeeding. No surgical technique or instrument in frenectomy is superior to another.

## Data availability statement

The authors declare that all data are available in repository.

## Declaration of competing interest

The authors declare no conflicts of interest.
