# Supplementary material for: Brazilian Academy of Paediatric Otorhinolaryngology Task Force – lingual frenulum disorders in childhood – evidence-based recommendations
Source: Braz J Otorhinolaryngol. 2026 Jan 17;92(2):101762. doi: 10.1016/j.bjorl.2026.101762 (PMC12854977; doi:10.1016/j.bjorl.2026.101762)
Supplement: Supplementary file 1 [file mmc1.docx]

**Appendix I** Methods for evaluating the lingual frenulum described in this article (in order of publication).


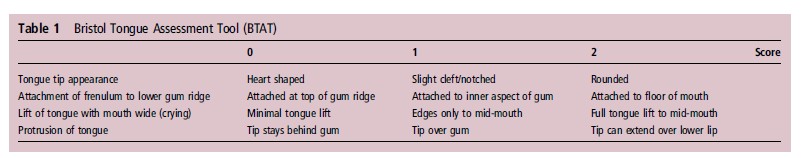


Bristol Tongue Asessment Tool

From: Ingram J, Johnson D, Copeland M, Churchill C, Taylor H, Emond A. The development of a tongue assessment tool to assist with tongue-tie identification. Arch Dis Child Fetal Neonatal Ed. 2015 Jul;100(4):F344-8. doi: 10.1136/archdischild-2014-307503. Epub 2015 Apr 15. PMID: 25877288; PMCID: PMC4484383.

TABBY Tongue Assessment Tool

From: Ingram J, Copeland M, Johnson D, Emond A. The development and evaluation of a picture tongue assessment tool for tongue-tie in breastfed babies (TABBY). Int Breastfeed J. 2019 Jul 16;14:31. doi: 10.1186/s13006-019-0224-y. PMID: 31346346; PMCID: PMC6636052.
